# Chronic kidney disease patients who smoke have higher serum phosphorus

**DOI:** 10.1590/2175-8239-JBN-2018-0156

**Published:** 2018-12-10

**Authors:** Geuza Dutra dos Santos, Rosilene Motta Elias, Maria Aparecida Dalboni, Giovânio Vieira da Silva, Rosa Maria Affonso Moysés

**Affiliations:** 1 Universidade Nove de Julho São PauloSP Brasil Universidade Nove de Julho, São Paulo, SP, Brasil.; 2 Hospital das Clínicas São PauloSP Brasil Hospital das Clínicas, São Paulo, SP, Brasil.

**Keywords:** Renal Insufficiency, Chronic, Chronic Kidney Disease-Mineral and Bone Disorder, Tobacco Use Disorder, Phosphorus

## Abstract

**Introduction:**

Mineral and bone metabolism disorders in chronic kidney disease (CKD-MBD) constitute a syndrome defined by changes in calcium, phosphorus (P), vitamin D and parathormone, fibroblast growth factor 23 (FGF-23) and its specific cofactor, Klotho. CKD-MBD, as well as smoking, are associated with an increased risk of cardiovascular disease. However, it is not known whether or not smoking impacts the cardiovascular risk in CKD- MBD.

**Objective:**

To analyze the relationship between smoking and CKD-MBD markers.

**Methods:**

We evaluated 92 patients divided into: 1) Control Group: non-smokers without CKD; 2) CKD group in stages III and IV under conservative treatment (20 non-smokers and 17 smokers); 3) CKD group on dialysis (21 non-smokers and 19 smokers). Clinical, demographic, and biochemical markers were compared between the groups.

**Results:**

FGF-23 and Klotho levels were not different between smokers and non-smokers. Patients in the CKD group on conservative treatment had higher serum P than non-smokers (*p* = 0.026) even after adjusted for renal function (*p* = 0.079), gender (*p* = 0.145) and age (*p* = 0.986).

**Conclusion:**

Smoking confers a higher cardiovascular risk to CKD patients under conservative treatment as it is associated with higher levels of P. Further studies are needed to confirm and better elucidate this finding.

## INTRODUCTION

Chronic kidney disease (CKD) is recognized as an independent risk factor for cardiovascular disease, which is the leading cause of death in this population. Mineral bone metabolism disorders and CKD (CKD-MBD) are a syndrome defined by changes in calcium (Ca), phosphorus (P), vitamin D and parathyroid hormone (PTH), bone abnormalities and extra skeletal calcification. They are common in patients with CKD, and are also an important cause of mortality and morbidity.^1^

Smoking, a classic cardiovascular risk factor, is associated with atherosclerosis, inflammation, increased progression of CKD^2^, and increased cardiovascular mortality in patients with CKD.^3^

Data from the literature show that smoking is associated with a higher risk of coronary calcification and higher levels of P in the general population.^4^ Thus, the objective of the present study was to analyze the relationship between smoking and traditional risk factors associated with CKD-MBD.

## METHODS

A cross-sectional study was conducted in patients attending the Nephrology Department of the Hospital das Clínicas of the São Paulo Medical School, FMUSP, from August 2016 to January 2017. Three groups of patients were included: 1) Control Group, consisting of patients with hypertension, non-smokers and without CKD; 2) Patients with CKD in stages III to IV (smokers and non-smokers); 3) Patients with CKD on dialysis (smokers and non-smokers).

We included patients aged 18 to 70 years, in regular follow-up at the outpatient clinic. Dialysis patients had been stable in the program for at least 6 months. Patients with diabetes mellitus, neoplasia, lupus, serology positive for HIV virus, as well as patients on immunosuppressants and corticosteroids were excluded. The study was approved by the Research Ethics Committee of the Nove de Julho University on June 4, 2016 (1,613,780).

We evaluated the patients' ages, genders, presence of comorbidities such as hypertension and history of coronary and cerebrovascular disease, medications used regularly and laboratory test results. Klotho dosages (IBL, Japan, sensitivity 6.15 pg/ml) were performed using enzyme-linked immunosorbent assay (ELISA), and those of FGF-23 with chemiluminescent assay (Diasorin, Italy, sensitivity 5.0 pg/ml).

## STATISTICAL ANALYSIS

Continuous data were expressed as mean and standard deviation or median and percentiles (25-75), according to parametric or non-parametric distribution, respectively. Categorical data were expressed as values and percentages. The comparison between the three groups was performed using ANOVA with Tukey or Kruskal-Wallis post-test, with Dunns post-test for variables with parametric or non-parametric distribution, respectively. The comparison between smokers and non-smokers was performed by unpaired t-test or Mann-Whitney, appropriately. The covariance analysis (ANCOVA) was performed with serum P as the dependent variable, eGFR and age as covariables, smoking and gender were fixed factors. The correlation between variables was made using the Spearman›s coefficient test. A *p* value < 0.05 was considered significant. Statistical analysis was performed using the SPSS 20.0 (SPSS Inc., Chicago, IL, USA).

## RESULTS

A total of 92 individuals were included, 15 in the Control group, 37 in the CKD group, and 40 in the CKD dialysis group (Table 1). The control group consisted mainly of female patients, who did not practice physical activity regularly. Patients in the CKD group on dialysis were younger. Differences in the Ca, P, PTH, creatinine and hemoglobin values are explained by the better renal function in the Control group. Uric acid levels were lower in the Control group when compared to patients with CKD under in conservative treatment. FGF-23 levels were higher in patients with CKD, and especially in those on dialysis when compared to patients in the Control group. We found correlation between FGF-23 levels and age (r = -0.258, *p* = 0.014), ferritin (r = 0.313, *p* = 0.011), phosphorus (r = 0.527, *p* = 0.0001) and PTH, *p* = 0.0001).

**Table 1:** Comparison between the demographical, clinical and laboratorial data among the three groups of patients

	Control	CKD conservative treatment		CKD in dialysis	
	N = 15	Non-smoker	Smoker	Non-smoker	Smoker
		N = 20	N = 17	N = 21	N = 19
Age (years)	56 ± 11	53 ± 13	53 ± 12	40 ± 13	50 ± 14 a
Males, %	13*	45	41	52	68
Physical activity, %	0*	60	12	24	16
EVA previous, %	0	0	0	4.8	0
CAF previous %	6.7	20	5.9 a	9.5	15.8
Creatinine (mg/dl)	0.8 (0.7-0.9)*	2.1 (1.7-2.9)	1.8 (1.4-3.55)	11.5 (9.4-14.45)	9.3 (8.3-11.3)
P (mg/dl)	3.6 ± 0.5*	3.6 ± 0.6	3.9 ± 0.5 a	4.8 ± 1.6	4.3 ± 1.3
Total Ca (mg/dl)	9.4 ± 0.3*	9.3 ± 1.1	9.1 ± 0.4	8.8 ± 0.8	8.8 ± 0.9
PTH (pg/ml)	53 (38-79)*	58 (45-95)	60 (34-101)	350 (82-765)	230 (151-358)
25 Vit-D (ng/dl)	21.8 ± 7.1	28.4 ± 9.8	30.9 ± 12.4	36.1 ± 15.3	26.8 ± 11.5 a
AP (U/l)	74 (61-86)	96 (68-112)	84 (68-94)	95 (63-129)	81 (64-132)
Uric acid (mg/dl)	5.0 (3.8-6.6)*	7.0 (6.2-8.1)	6.5 (5.6-8.1)	6.3 (5.6-8.0)	5.7 (4.8-6.4)
Hemoglobin (g/dl)	13.6 ± 1.1*	12.7 ± 1.4	12.7 ± 2.3	10.9 ± 1.5	10.9 ±1.6
Albumin (g/dl)	-	4.3 (3.9-4.5)	4.05 (3.5-4.7)	3.8 (3.4-4.1)	3.9 (3.6-4.2)
Ferritin (ng/ml)	-	157.1 (113.2-213.5)	132.2 (87-197)	333.9 (116-610)	345 (145-636)
eGFR (mL/min/1.73m ^2^)	89 (77-104)*	28 (21-43)	38 (17-50)	-	-
FGF-23 (pg/ml)	76.7 (58.7-90.7)*	92.5 (69.3-287.6)	141.2 (104.9-222.2)	5208 (284-18257)	474.5 (116-5800)

EVA: Stroke; CAF: coronary artery failure; P: phosphorus; Total Ca: Total calcium; PTH: Parathyroid hormone; 25 Vit D: 25 Hydroxyvitamin D; AP: Alkaline phosphatase; Sat: saturation; eGFR: Estimated Glomerular Filtration Rate; FGF-23: Fibroblast growth factor23.

* *p* < 0.05 vs. CKD a: *p* < 00.5 *vs*. Non-smoker in the same group.

Regarding medications in use, patients in the Control group received less vitamin D supplementation; whereas patients under conservative CKD treatment more commonly used furosemide, and dialysis patients received more calcitriol, calcium salts, Sevelamer, and human erythropoietin (EPO).

**Figure 1:**
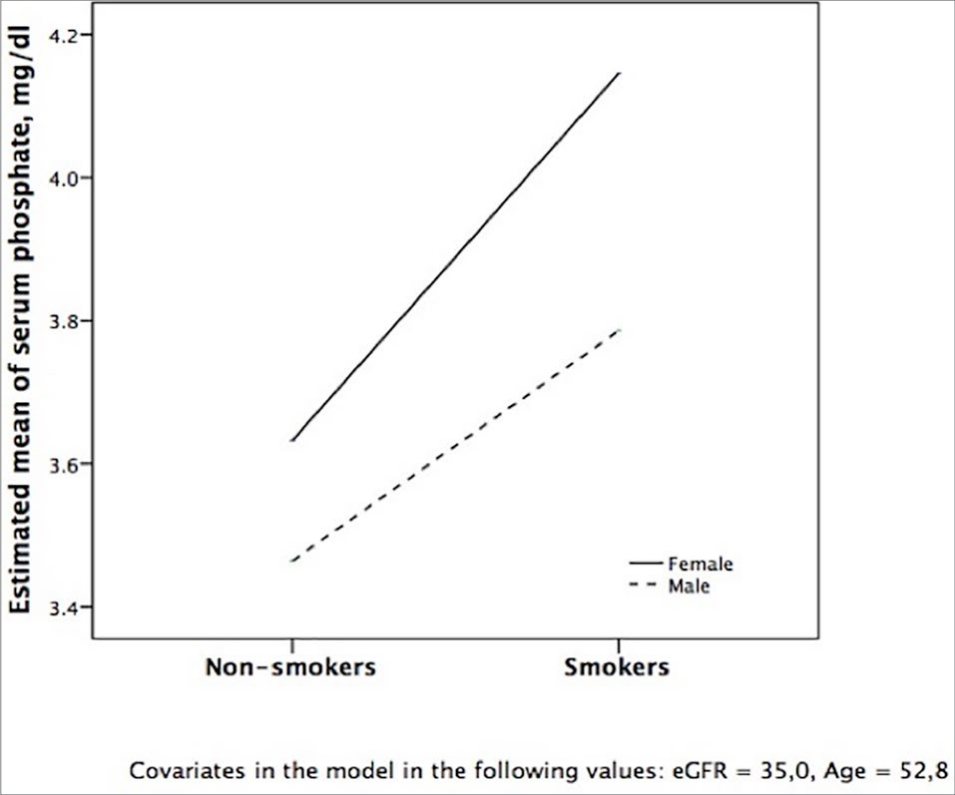
Phosphorus levels between smokers and non-smokers among CKD patients under conservative treatment according to gender, adjusting for kidney function and age in a covariance analysis. The full line represents females and the dotted line represents males.

Comparing smokers and non-smokers (Table 1), we found that smokers on dialysis were younger and had lower levels of vitamin D. Levels of FGF-23 and Klotho were not different between smokers and non-smokers. Smoking patients in the CKD group under conservative treatment had higher serum P than non-smokers (Table 1). P levels remained higher in these patients (*p* = 0.026), even after adjusting for eGFR (*p* = 0.079), gender (*p* = 0.145) and age (*p* = 0.986) in a model with adjusted R2 of 0.140, as demonstrated in Figure 1.

## DISCUSSION

Comparing smoking and non-smoking CKD patients, we observed that smoking was associated with higher levels of P in those receiving conservative treatment. The highest P in smokers was independent of gender, FGF-23 and renal function. No other CKD-MBD marker differed between smokers and non-smokers.

We found correlations between serum FGF-23 levels with age, phosphorus, PTH and ferritin. As individuals age, FGF-23 falls. On the other hand, as previously described by other authors, FGF-23 levels increase with the progression of CKD.^5^ Likewise, there was a positive association between FGF-23 with P and PTH, according to Lavi-Moshayoff et al., who demonstrated that PTH enhances FGF-23.^6^ gene transcription. The relationship between FGF-23 and ferritin was inverse to that reported in the literature.^7^ However, experimental studies have shown that both iron deficiency and administration of some iron-rich compounds can stimulate the synthesis of FGF-23.^8^

We found that serum phosphorus was higher in the group of smokers under conservative treatment. Recalling that serum phosphorus has already been identified as an independent risk factor for mortality due to cardiovascular etiology among chronic renal individuals,^3^ it is curious to note that smokers with higher cardiovascular risk also have higher serum phosphorus, even within the reference range. One possibility would be that smoking inhibited Klotho's renal expression (and therefore its serum level), decreasing FGF-23 action. Thus, although with similar values of FGF-23, smokers would have lower phosphaturic action of this hormone and, therefore, higher serum levels of phosphorus. However, we did not find differences in serum Klotho levels between the two groups. The current concept is that in CKD there is an important suppression of Klotho transcription in the renal tissue. Using the Western-Blot technique, Kuro-o et al. showed a decrease in the urinary concentration of Klotho as CKD progresses.^9^ However, it is important to note that we evaluated the serum concentration of Klotho using the ELISA technique. Currently, there is still controversy as to whether serum Klotho levels increase or decrease with the progression of CKD, and whether ELISA assays are reliable.^10^ Nor have we found the impact of smoking on FGF-23 levels, contrary to what has been described previously.^11^ This result could be explained by the use of different assays, since we measured the intact molecule and these authors evaluated the C-terminal fraction of FGF-23. This fragment of the FGF-23 molecule, previously identified as inactive, appears to play an important role in iron deficiency and inflammation.^8^ However, the role of C-terminal FGF-23 under these conditions is poorly understood. Interestingly, epidemiological studies have shown a negative association between smoking and Parkinson's disease,^12^ so that smokers had higher serum P and had a lower risk of the disease. Higher P levels were also observed in men and women with diabetes, even after adjusting for age and other cardiovascular risk factors.^13^ The authors suggested that smokers may have greater bone resorption and/or less bone mineralization, leading to a higher P, which was previously demonstrated.^14^ As far as we could see, no data was found in the CKD population. However, in patients with primary hyperparathyroidism, smoking has been shown to be associated with higher P levels and lower PTH levels.^15^

For dialysis patients, there were no differences in serum phosphorus between smokers and non-smokers. Probably, in this group of patients, dialysis, PTH and the use of binders should be the main determinants of serum phosphorus.

In summary, smoking, in addition to conferring an increase in cardiovascular risk alone in the population of patients with CKD, is associated with higher levels of P in patients on conservative treatment, which confers an additional risk of mortality. The reasons for this finding remain unclear, but it appears to be independent of renal function, gender, age, and FGF-23.

We recognize the limitations of this study in view of the relatively small sample size and cross-sectional design, which does not enable us to establish cause and effect relationships. However, this is an exploratory study of a hypothesis, and new studies are needed to explore the relationship between P and smoking and to elucidate the mechanism involved in this association.

## References

[1] Moe S, Drüeke T, Cunningham J, Goodman W, Martin K, Olgaard K, et al.; Kidney Disease: Improving Global Outcomes (KDIGO). Definition, evaluation, and classification of renal osteodystrophy: a position statement from Kidney Disease: Improving Global Outcomes (KDIGO). Kidney Int 2006;69:1945-53. 10.1038/sj.ki.5000414 16641930

[2] Nagasawa Y, Yamamoto R, Rakugi H, Isaka Y. Cigarette smoking and chronic kidney diseases. Hypertens Res 2012;35:261-5. DOI: 10.1038/hr.2011.205 10.1038/hr.2011.205 22158113

[3] Nakamura K, Nakagawa H, Murakami Y, Kitamura A, Kiyama M, Sakata K, et al.; EPOCH-JAPAN research group. Smoking increases the risk of all-cause and cardiovascular mortality in patients with chronic kidney disease. Kidney Int 2015;88:1144-52. DOI: 10.1038/ki.2015.212 10.1038/ki.2015.212 26200944

[4] Lehmann N, Möhlenkamp S, Mahabadi AA, Schmermund A, Roggenbuck U, Seibel R, et al. Effect of smoking and other traditional risk factors on the onset of coronary artery calcification: results of the Heinz Nixdorf recall study. Atherosclerosis 2014;232:339-45. DOI: 10.1016/j.atherosclerosis.2013.11.045 10.1016/j.atherosclerosis.2013.11.045 24468147

[5] Oliveira RB, Moysés RM. FGF-23: state of the art. J Bras Nefrol 2010;32:323-31. 21103697

[6] Lavi-Moshayoff V, Wasserman G, Meir T, Silver J, Naveh-Many T. PTH increases FGF23 gene expression and mediates the high-FGF23 levels of experimental kidney failure: a bone parathyroid feedback loop. Am J Physiol Renal Physiol 2010;299:F882-9. DOI: 10.1152/ajprenal.00360.2010 10.1152/ajprenal.00360.2010 20685823

[7] Honda H, Michihata T, Shishido K, Takahashi K, Takahashi G, Hosaka N, et al. High fibroblast growth factor 23 levels are associated with decreased ferritin levels and increased intravenous iron doses in hemodialysis patients. PLoS One 2017;12:e0176984. DOI: 10.1371/journal.pone.0176984 10.1371/journal.pone.0176984 PMC5419608 28475601

[8] Francis C, David V. Inflammation regulates fibroblast growth factor 23 production. Curr Opin Nephrol Hypertens 2016;25:325-32. DOI: 10.1097/MNH.0000000000000232 10.1097/MNH.0000000000000232 PMC5016608 27191351

[9] Hu MC, Kuro-o M, Moe OW. Secreted klotho and chronic kidney disease. Adv Exp Med Biol 2012;728:126-57. DOI: 10.1007/978-1-4614-0887-1_9 10.1007/978-1-4614-0887-1_9 PMC3780390 22396167

[10] Maxwell PH. Hypoxia-inducible factor as a physiological regulator. Exp Physiol 2005;90:791-7. 10.1113/expphysiol.2005.030924 16157658

[11] Vervloet MG, van Zuilen AD, Heijboer AC, ter Wee PM, Bots ML, Blankestijn PJ, et al. Fibroblast growth factor 23 is associated with proteinuria and smoking in chronic kidney disease: an analysis of the MASTERPLAN cohort. BMC Nephrol 2012;13:20. DOI: 10.1186/1471-2369-13-20 10.1186/1471-2369-13-20 PMC3366907 22530966

[12] Håglin L. High serum phosphate concentration as the result of smoking might underlie the lower risk of Parkinson's disease. Med Hypotheses 2015;85:287-90. DOI: 10.1016/j.mehy.2015.05.017 10.1016/j.mehy.2015.05.017 26206759

[13] Håglin LM, Törnkvist B, Bäckman LO. High serum phosphate and triglyceride levels in smoking women and men with CVD risk and type 2 diabetes. Diabetol Metab Syndr 2014;6:39. DOI: 10.1186/1758-5996-6-39 10.1186/1758-5996-6-39 PMC3995531 24636522

[14] Krall EA, Dawson-Hughes B. Smoking increases bone loss and decreases intestinal calcium absorption. J Bone Miner Res 1999;14:215-20. 10.1359/jbmr.1999.14.2.215 9933475

[15] Amstrup AK, Rejnmark L, Vestergaard P, Heickendorff L, Mosekilde L. Effects of smoking on severity of disease in primary hyperparathyroidism. Calcif Tissue Int 2010;87:406-13. DOI: 10.1007/s00223-010-9416-6 10.1007/s00223-010-9416-6 20862465

